# CRISPR/Cas9 Landscape: Current State and Future Perspectives

**DOI:** 10.3390/ijms242216077

**Published:** 2023-11-08

**Authors:** Marina Tyumentseva, Aleksandr Tyumentsev, Vasiliy Akimkin

**Affiliations:** Central Research Institute of Epidemiology, Novogireevskaya Str., 3a, 111123 Moscow, Russia; tymencev@cmd.su (A.T.); akimkin@pcr.ms (V.A.)

**Keywords:** genome editing, CRISPR/Cas9, therapeutics, clinical trials, diagnostics, Cas9 orthologs

## Abstract

CRISPR (clustered regularly interspaced short palindromic repeats)/Cas9 is a unique genome editing tool that can be easily used in a wide range of applications, including functional genomics, transcriptomics, epigenetics, biotechnology, plant engineering, livestock breeding, gene therapy, diagnostics, and so on. This review is focused on the current CRISPR/Cas9 landscape, e.g., on Cas9 variants with improved properties, on Cas9-derived and fusion proteins, on Cas9 delivery methods, on pre-existing immunity against CRISPR/Cas9 proteins, anti-CRISPR proteins, and their possible roles in CRISPR/Cas9 function improvement. Moreover, this review presents a detailed outline of CRISPR/Cas9-based diagnostics and therapeutic approaches. Finally, the review addresses the future expansion of genome editors’ toolbox with Cas9 orthologs and other CRISPR/Cas proteins.

## 1. Introduction

Genome editing has taken a leading position among genome modification technologies in a short time and is now widely used in gene therapy. To date, there are three main systems for genome editing: zinc finger nucleases (ZFNs), transcription activator-like effector nucleases (TALENs), and CRISPR/Cas nucleases. Genome editing has been successfully used in the field of functional genomics for the identification of the function of genes and genetic elements that regulate gene expression and for deciphering the mechanisms of cross-talk of gene function in the cell. In addition, programmable nucleases are often used to validate human disease-associated genes and to create gene knockouts in a variety of cell lines. Moreover, the ability to provide a complete knockout of genes that are not amenable to RNA interference, another common method of functional genomics, can be considered an important achievement of the use of programmable nucleases [1,2,3].

In addition to functional genomics, programmable nucleases have been successfully used for cell screening, which allows the development of modified cell lines with inserted promoters, labels, or reporter elements integrated into endogenous genes or intergenic regions [4]. Often, programmable nucleases are used to develop and optimize cell lines with desired properties, for example, superexpressors of recombinant proteins or antibodies for biotechnological and pharmacological purposes [5,6].

Since 2009, when the first knockout rat was developed [7], programmable nucleases have been successfully applied at the level of whole organisms, most often to establish animal models of human diseases and to improve plant varieties and breeds of farm animals [8,9,10,11,12,13].

**Figure 1:**
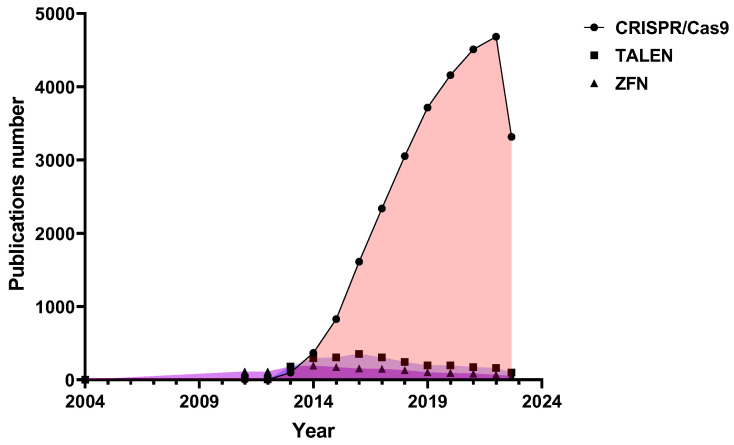
The number of articles in PubMed^®^ (https://pubmed.ncbi.nlm.nih.gov/, accessed on 5 September 2023) with search terms “CRISPR Cas9”, “TALEN”, and “zinc finger nuclease”.

Scientific interest in programmable nucleases has only been growing in the last decade, and most often there are scientific papers devoted to the development and study of CRISPR/Cas9 nucleases (Figure 1). Last year marked 10 years since the development of CRISPR/Cas9 as a genome editing tool, and Jennifer Doudna and Emmanuelle Charpentier were awarded the 2020 Nobel Prize in Chemistry for discovering one of gene technology’s sharpest tools: the CRISPR/Cas9 genetic scissors.

Finally, programmable nucleases are used to develop therapeutic drugs. In 2009, the first clinical trial, NCT00842634 (https://clinicaltrials.gov/study/NCT00842634, accessed on 5 September 2023), of a candidate therapeutic drug based on zinc finger nucleases was initiated; in 2016, based on CRISPR/Cas (NCT02793856-https://clinicaltrials.gov/study/NCT02793856, accessed on 5 September 2023, NCT02867345-https://clinicaltrials.gov/study/NCT02867345, accessed on 5 September 2023, NCT02863913-https://clinicaltrials.gov/study/NCT02863913, accessed on 5 September 2023, and NCT02867332-https://clinicaltrials.gov/study/NCT02867332, accessed on 5 September 2023); and in 2017-based on TALENs (NCT03226470-https://clinicaltrials.gov/study/NCT03226470, accessed on 5 September 2023).

**Figure 2:**
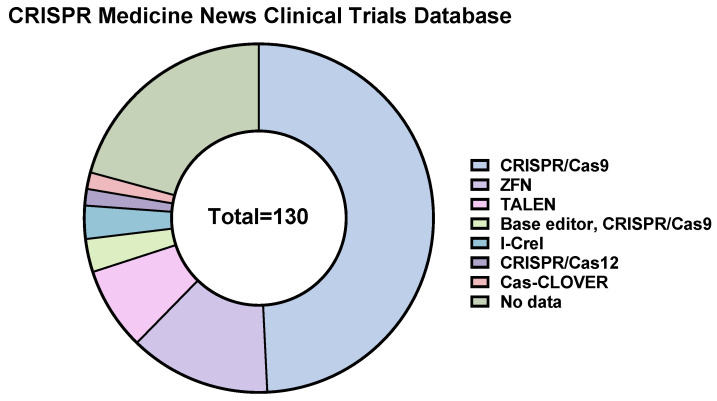
The number of genome editing clinical trials mentioned on the CRISPR Medicine News website (https://crisprmedicinenews.com/clinical-trials/, accessed on 5 September 2023).

To date, 130 genome editing clinical trials are mentioned on the CRISPR Medicine News website (https://crisprmedicinenews.com/clinical-trials/, accessed on 5 September 2023). Of them, ~50% are clinical trials of CRISPR/Cas9-based therapeutics (Figure 2).

From the very beginning, the CRISPR/Cas9-based therapeutic approach was the most promising. 10 years seems like a short time in the innovative drug discovery process, but CRISPR-based therapies have made significant progress. For the first five years, researchers have been modifying existing CRISPR/Cas9 proteins to achieve increased genome editing efficiency and reduced off-target activity and developing CRISPR for clinical use for the first time. Over the next five years, new CRISPR/Cas proteins with different capabilities were discovered and developed, the CRISPR toolbox expanded, and the first CRISPR/Cas9 trials were conducted, sometimes with amazing results.

In this review article, we will focus on the current CRISPR/Cas9 landscape. We will discuss Cas9 variants with improved efficacy and specificity, Cas9 *S. pyogenes* nickases and catalytically inactive Cas9 *S. pyogenes* (dCas9), Cas9-derived proteins such as base editors, prime editors, dCas9-based imaging tools, Cas9-based transcription repressors and activators, and Cas9-fusion proteins such as Cas-CLOVER and Cas-FokI. Moreover, we will talk about CRISPR/Cas9 delivery methods, pre-existing immunity against CRISPR/Cas9 proteins, anti-CRISPR proteins, and their possible role in CRISPR/Cas9 efficacy improvement. We will discuss CRISPR/Cas9-based diagnostics and therapeutic approaches. Finally, we will address future perspectives on CRISPR/Cas9 genome editing and the expansion of genome editors’ toolboxes with Cas9 orthologs, other CRISPR/Cas proteins, and recently described Fanzor enzymes.

## 2. Cas9 *S. pyogenes*—The Most Widely Used Cas9 Nuclease

Nowadays, Cas9 from *S. pyogenes* (SpCas9 or SpyCas9) is the most common, well-known multi-domain CRISPR/Cas effector protein widely used for genome editing. SpyCas9 is an RNA-dependent DNA endonuclease consisting of two nuclease domains—RuvC and HNH, which are responsible for inducing a double-strand break in the target DNA sequence. Different types of guide RNA (gRNA) are used for SpyCas9-specific targeting. For example, the guide RNA can represent a complex of CRISPR RNA (crRNA) responsible for specific recognition of the target sequence and trans-activating CRISPR RNA (tracrRNA) responsible for the binding of the enzyme and essential for pre-crRNA processing. In addition, the guide RNA can be a single guide RNA (sgRNA), which combines crRNA and tracrRNA in a single molecule [14].

The off-target activity of SpyCas9 still remains a problem, as Cas9 can edit a DNA-target carrying up to five mismatches with its guide RNA [15,16,17]. This off-target effect has been extensively analyzed by various in vitro and in vivo approaches [18,19,20], and the criteria for specificity of the SpyCas9 system can be described as follows: (1) In most cases, the system cannot recognize a DNA site carrying more than three mismatches; (2) the CRISPR/Cas9 system cannot recognize and edit a DNA site with any number of mismatches adjacent to protospacer adjacent motif–PAM (within 10–12 bp); (3) the higher the concentration of the CRISPR/Cas9 complex, the higher the likelihood of non-specific activity; (4) some 5′-NAG-3′-PAM sites can be targeted by the CRISPR/Cas9 system in bacteria and in vitro experiments, but Cas9 has a much lower affinity for NAG-PAM than for NGG-PAM. In addition, next-generation sequencing-based methods such as genome-wide, unbiased identification of double-strand breaks enabled by sequencing (GUIDE-seq) [21], digested genome sequencing (Digenome-seq) [22], and Chromatin Immunoprecipitation Sequencing (ChIP-seq) [23] can identify non-target sites for the CRISPR/Cas9 system. These high-throughput assays have confirmed that Cas9 has off-target activity, and careful guide RNA design is necessary to reduce the risk of the off-target activity of Cas9.

The approaches used to diminish off-target effects include engineering SpyCas9 variants with improved efficacy and specificity [15,24,25,26,27,28,29,30,31,32,33,34,35,36,37,38,39,40,41], using SpyCas9 nickases [35], base [42,43,44,45,46,47,48,49,50,51] and prime editors [52,53,54], Cas-CLOVER [55,56,57], and Cas-FokI [41,58,59,60,61,62]. As well, SpyCas9 off-target activity can be reduced through optimization and/or modulation of gRNA design [63,64,65,66,67,68,69,70,71] and combination with anti-CRISPR proteins or CRISPR inhibitors [72,73,74,75]. Moreover, Cas9 orthologs with high specificity can be used instead of SpyCas9 [76,77].

## 3. Cas9 Variants with Improved Efficacy and Specificity

The problem of nonspecific editing is being solved by constructing (engineering) new variants of *S. pyogenes* Cas9 [15,24,25,26,27,28,29,30,31,32,33,34,35,36,37,38,39,40,41]. Improved SpCas9 variants were generated using both methods of directed evolution (i.e., random mutagenesis combined with high-throughput screening) and structure- and/or function-guided protein engineering approaches.

Besides the high-fidelity SpCas9 variants, other Cas9 proteins, such as *Staphylococcus aureus* Cas9 (SaCas9), with different PAM requirements, were developed. SaCas9 has comparably high activity in eukaryotic cells [78,79,80] and has a more compact size (1053 amino acids for SaCas9 versus 1368 amino acids for SpCas9). Several high-fidelity SaCas9 variants were constructed. All these variants possessed high on-target activities and comparable specificity at most off-target loci [27,28,32,39]. Also, high-fidelity SaCas9 variants with PAM-altered or -relaxed mutations were reported [81,82,83].

All the Cas9 variants mentioned above are listed in the table below (Table 1) and form a versatile toolbox of high-fidelity Cas9 proteins. But still, more Cas9 variants with both high fidelity and activity need to be designed and investigated for further therapeutic applications in vivo.

## 4. Cas9 *S. pyogenes* Nickases

By introducing mutations into one of the two SpyCas9 nuclease domains, researchers developed SpyCas9 nickases. The D10A mutation inactivates the RuvC nuclease domain, so this nickase only cleaves the target strand (which is complementary to sgRNA). On the other hand, the H840A mutation in the HNH–nuclease domain cleaves the second strand [87]. The main feature of nickase is that it introduces a single-strand break in the DNA. It has been shown that genome editing with the simultaneous use of two guide RNAs and SpyCas9 nickase reduces the likelihood of nonspecific editing. As single-strand breaks are usually rapidly repaired by homologous recombination with an intact complementary template DNA strand, the off-target effects of SpyCas9 nickases are minimized [35].

Cas9 nickases introduce tandem single-strand DNA breaks at target loci and, together with exogenous donor DNA templates, foster seamless and scarless genome editing through homology-mediated end joining (HMEJ). SpyCas9D10A nickase does not trigger p53 signaling in human iPSCs, which makes it a fitting tool for the genomic engineering of cells with high sensitivity to DNA damage, e.g., pluripotent and tissue-specific stem cells [88].

In 2021, the existing Cas9 nickase toolkit was enlarged by the conversion of a panel of Cas9 nucleases with enhanced specificities, i.e., SpCas9-KA [27], SpCas9-KARA [27], eSpCas9 (1.1) [27], Sniper-Cas9 [33], SpCas9-HF1 [24], evoCas9 [29], and xCas9-3.7 [38], into Cas9 nickases [89]. These high-specificity Cas9 nickases are capable of distinguishing highly similar sequences and preserving genomic integrity, as demonstrated by unbiased genome-wide high-throughput sequencing assays [89].

The existing CRISPR/Cas9 nickase toolkit expands the range and precision of DNA knockout and knock-in procedures. CRISPR/Cas9 nickases may help to develop novel high-specificity genome editing therapeutic strategies with improved predictability and safety.

## 5. Catalytically Inactive Cas9 *S. pyogenes* (dCas9) and Its Derivatives

### 5.1. dCas9 S. pyogenes

In 2013, Qi et al. performed mutagenesis in the catalytic nuclease domains of the Cas9 protein from *S. pyogenes*. As a result, two mutations were introduced: H840A in the HNH domain and D10A in the RuvC domain of Cas9. Thus, a catalytically inactive Cas9 protein, also called “dead Cas9” or dCas9 null mutant, was obtained [90]. dCas9 is unable to cleave target DNA but retains the ability to RNA-mediated (sgRNA) target DNA sequence binding with the same precision as catalytically active Cas9. Unlike Cas9, dCas9 does not cause irreversible changes in the genome but only affects the transcription of the target site, which leads to reversible gene silencing.

dCas9 bound to a target site causes steric hindrance, which interferes with the normal functioning of the transcription apparatus. This effect underlies a method called CRISPR interference (CRISPRi). CRISPRi provides a simplified approach for rapid gene repression that can be used in a wide variety of organisms and can be adapted for high-throughput interrogation of genome-wide gene functions and genetic interactions [91]. CRISPRi repression efficiency is rather high (up to 1000-fold) with no detectable off-target effects [92]. It works quite well in bacterial, yeast, and other prokaryotic cells but is less effective at suppressing gene expression in mammalian cells [93]. What is more, CRISPRi can be easily scaled up for the simultaneous repression of many genes using multiple designed guide RNAs [94].

As CRISPRi-mediated genetic screens have the potential to address basic questions in cell biology, genetics, and biotechnology, sgRNA libraries are established to enable strong and consistent knockdown across mammalian cell lines [95]. Moreover, the CRISPRi system can be fine-tuned to regulate gene expression at a certain level between fully-repressing and non-repressing states (>45-fold) to optimize processes in metabolic engineering and synthetic biology [96].

Using dCas9 itself as a transcription repressor was just the beginning. Soon after, researchers began creating chimeric dCas9s with the effector domains of repressor or activator proteins to exploit dCas9’s targeting capabilities for reversible gene activation, epigenomic editing, and more. Whether it’s a promoter region, a regulatory region, or a coding region, scientists can use dCas9 as a modular scaffold to easily attach an effector, allowing control of any gene without introducing irreversible DNA-damaging mutations.

### 5.2. dCas9-Based Transcription Repressors

Limitations in the use of CRISPRi led to the development of the dCas9-KRAB system, in which dCas9 is fused with a functional KRAB (Krüppel-associated box) transcriptional repressor domain [93]. This system mediates transcriptional repression through the ability of KRAB to recruit a diverse set of histone modifiers that reversibly suppress gene expression through the formation of heterochromatin. Using this system, a 60–80% reduction in the expression of highly specific eukaryotic genes was achieved during transient transfection [93]. In addition, dCas9-KRAB, stably integrated into the genome in HeLa cells, caused a stable 5–10-fold repression of genes and promoter regions [93], and a 100-fold effect was observed when the target site was located at 50–100 bp downstream of the transcription start site [90]. The presence of dCas9-KRAB also had no effect on cell growth or viability [97]. What is more, the dCas9-KRAB repressor can be induced by adding abscisic and gibberellic acids [98], and doxycycline [99,100].

Unlike other classical approaches to gene inactivation, such as RNA interference, the dCas9-KRAB system provides reversible inhibition at the DNA level [101]. This provides highly specific gene repression as well as inhibition of non-coding RNAs, microRNAs, antisense transcripts, and nuclear-localized RNAs [93].

dCas9-based repression is a powerful platform for silencing gene expression, but it suffers from incomplete silencing of target genes; therefore, novel highly efficient repressors had to be developed. In 2018, Nan Cher Yeo et al. showed that dCas9 tandemly fused with KRAB-MeCP2 demonstrated improved gene silencing when compared to dCas9-KRAB [102]. Later, the dCas9-KRAB toolkit was expanded with 57 KRAB domains with different potencies. The ZIM3 KRAB domain was identified as an exceptionally potent repressor that silences gene expression more efficiently than existing platforms [103]. It should be mentioned that ZIM3-dCas9 provides an excellent balance between strong on-target knockdown and minimal non-specific effects on cell growth or the transcriptome [95]. Recently, dCas9-SALL1-SDS3 was shown to significantly improve CRISPR-mediated repression compared to dCas9-KRAB and dCas9-KRAB-MeCP2. dCas9-SALL1-SDS3 exhibits higher levels of target gene repression while retaining high target specificity [104].

What is more, dSaCas9-KRAB repressors expand the CRISPR/Cas9 toolbox for basic research and gene therapy applications [105], and dCas9-SRDX repressors are used for silencing gene expression in plants [106,107,108].

dCas9-based transcription repressors allow researchers to selectively block the expression of target genes in their natural chromosomal context. In combination with sgRNA libraries, dCas9-based transcription repressors can be easily used for large-scale functional genome screening studies.

### 5.3. dCas9-Based Transcription Activators

To endow dCas9 with the ability to activate genes, dCas9 was fused to classical transcription activators such as VP64 (a synthetic tetramer of the herpes simplex virus 16 protein) or p65 (a transcription factor involved in many cellular processes). Although these systems have demonstrated the ability to activate gene transcription in a variety of eukaryotic cells, only moderate activation (2- to 5-fold) has been achieved [109].

In order to increase the degree of activation, a system of synergistic activation mediators (SAM) was developed [110]. This system is based on dCas9-VP64 but includes modified sgRNA able to recruit additional transcriptional activators to achieve a synergistic activation effect. This modified sgRNA contains two hairpin RNA aptamers that bind dimers of bacteriophage MS2 coat proteins. Fusion of bacteriophage MS2 coat proteins with additional activators such as p65 and human heat shock factor 1 (HSF1) results in the recruitment of 13 activating molecules per dCas9 molecule. This novel dCas9-SAM system can reliably enhance gene expression from 10- to several thousand folds, depending on the level of basal expression [109].

Another dCas9-based system for transcription activation uses a novel protein scaffold, a repeating peptide array termed SunTag, which can recruit multiple copies of VP64. This system allows for strong activation of endogenous gene expression [111,112]. To further improve the efficiency of dCas9-based transcription activators, the dCas9-SPH (SPH stands for SunTag with p65-HSF1) platform was established [113].

Church and colleagues developed a system that consists of a combination of three activators, VP64, p65, and RTA, the so-called dCas9-VPR system [114]. Effective activation with dCas9-VPR does not require the use of aptamer-modified sgRNA, greatly simplifying the development process. The degree of gene activation achieved through the use of the proposed system was comparable to that using dCas9-SAM [115].

dCas9-based transcription activators can become an indispensable tool in the development of therapeutic drugs for genetic screening and transcriptional manipulation of endogenous and synthetic genetic sequences in various cell types [92,115]. Researchers are already using the dCas9-SAM system to activate human immunodeficiency virus 1 (HIV-1) transcription to induce apoptosis and subsequent destruction of infected cells, as well as to induce transcription of HIV-1 proviral DNA integrated into the genome of cellular reservoirs for its complete elimination [116,117].

Moreover, dCas9-VPR can be used to develop novel, attractive gene therapies for inherited disorders caused by mutations in disease-causing genes that possess functionally equivalent counterparts. dCas9-VPR is used to transcriptionally activate the appropriate counterpart(s) to compensate for the missing gene function. The main advantage of such an approach is that it is mutation- and gene-size-independent, unlike conventional gene therapies [118].

dCas9-based transcription activators [119] are simple tools that allow researchers to selectively activate the expression of target genes in their natural chromosomal context. When combined with a sgRNA library, dCas9-based transcription activators can be used for large-scale functional screening studies, making them a powerful tool for studying biological processes and signaling pathways.

### 5.4. dCas9-Mediated Epigenetic Editing

It is well known that the phenotype can be influenced to varying degrees by epigenetic modifications, which include modifications of both nucleosomes and the DNA itself. Epigenetic regulation affects the structure of regions of chromatin, either compressing it into a compact and transcriptionally inactive state (heterochromatin) or opening it for expression (euchromatin). Years of effort in functional genomics have mapped and characterized millions of epigenetic regulatory elements in a variety of tissues and cell types, but current methods for studying each locus are labor-intensive, expensive, and can be toxic to living cells. To study the epigenome, chimeric dCas9s with different effector domains were generated.

Histone acetylation is one of the most powerful systems that enhances gene expression. Hilton et al. developed the dCas9-p300 system, which allows direct modification of the chromatin state involved in a wide range of cellular pathways and processes [120]. In this system, dCas9 is combined with the catalytic domain of the human E1A-associated protein p300, a key component that acetylates histones. dCas9-p300 successfully activates gene expression when targeting coding or regulatory regions, demonstrating its effectiveness as a transactivator of downstream genes [120]. Activation ranges from 50- to 10,000-fold when targeting promoters or enhancers and is highly specific, as confirmed by transcriptome profiling [120]. dCas9-p300 uses mammalian p300, so it has minimal immunogenicity potential, making it attractive for in vivo applications. Recently, dCas9-p300 was used to facilitate human foreskin fibroblast transdifferentiation into Leydig-like cells to treat male hypogonadism [121]. Interestingly, dCas9-p300 is able to increase histone acetylation at the enhancer region of the activity-regulated cytoskeleton-associated protein (Arc) immediate-early gene, known as synaptic activity response element (SARE), normalizing deficits in Arc expression, leading to attenuation of adult anxiety and excessive alcohol drinking in a rat model of adolescent alcohol exposure [122].

dCas9-LSD1 is a gene repression system in which dCas9 is combined with lysine-specific histone demethylase 1 (LSD1). dCas9-LSD1 has the ability to repress downstream genes by targeting the distal enhancer region of a gene but not its promoter, making dCas9-LSD1 a promising tool for studying the regulatory activity of enhancers [123,124]. 

The dCas9-TET1CD system is capable of editing the epigenome through targeted demethylation. In this system, catalytically inactive Cas9 is combined with the catalytic domain (CD, catalytic domain) of the protein TET1 (Tet Methylcytosine Dioxygenase 1), an enzyme that triggers DNA demethylation. The guide RNA can be further modified to recruit bacteriophage MS2 coat proteins, which additionally carry two more TET1CD modules each. Such a system has demonstrated the ability to increase transcription across an array of genes in a locus-specific manner with little off-target variation in different human and mouse cell lines [125]. The dCas9-TET1CD system was successfully used for epigenetic editing in the promoter region of the tumor suppressor gene *BRCA1*, whose excessive repression through hypermethylation is associated with the occurrence of breast and ovarian cancer [126]. Also, the dCas9-TET1CD demethylation system can target spontaneous epialleles in Arabidopsis, leading to methylation reduction and being stably inherited in the progeny. These findings may give rise to future research on spontaneous epialleles in other crops and also broaden ideas for future crop breeding [127].

dCas9-DNMT3A (DNA methyltransferase 3 alpha), developed by Vojta et al., combines, via a flexible glycine-serine linker (Gly4Ser), dCas9 with the catalytic domain of DNMT3A, an active DNA methyltransferase that is capable of methylating CpG sites in vivo. dCas9-DNMT3A was shown to successfully induce site-specific CpG methylation distal and proximal to the promoter, with the highest methylation activity (60%) at 27 bp below the PAM sequence. When multiple guide RNAs were used, the effect of dCas9-DNMT3A was synergistic. dCas9-DNMT3A has also been used to directly methylate the promoters of tumor suppressor genes, the hypermethylation of which has been correlated with the occurrence of several types of cancer [128]. In 2022, it was shown that targeted methylation of the amyloid precursor protein gene via dCas9-DNMT3A can be a potential therapeutic strategy for Alzheimer’s disease [129]. To improve dCas9-DNMT3A activity, a modular dCas9-SunTag-DNMT3A epigenome editing system was developed. dCas9-SunTag-DNMT3A overcomes the pervasive off-target activity of dCas9-DNMT3A constructs [130].

In 2021, a programmable epigenetic memory writer consisting of a single catalytically inactive Cas9 fusion protein that establishes DNA methylation and repressive histone modifications named CRISPRoff was presented. The main advantages of CRISPRoff are (i) its ability to silence most genes, including those without CpG islands; (ii) its high specificity and broad targeting window across gene promoters; and (iii) its persistence of epigenetic memory through the differentiation process [131].

dCas9-fusion proteins for transcriptional and epigenetic regulation are extremely diverse (see Table 2 below) and allow mapping the complex relationships between the epigenome, regulatory elements, and target gene expression in functional genomics studies. The use of such tools in combination with guide RNA libraries may provide a high-throughput and systematic way to identify all enhancers and suppressors associated with genes of interest and study enhancer (suppressor)-gene interactions. Also, these tools will aid in the investigation of the role of DNA methylation in gene expression regulation in specific genomic contexts. Such systems could also be used to restore the functional activity of other tumor suppressor genes needed to fight cancer and other diseases.

### 5.5. dCas9-Based Imaging Tools

In addition to epigenetic editing applications, catalytically inactive Cas9 fused to a fluorescent marker such as green fluorescent protein (GFP) can be used to visualize genomic loci in living cells in vitro as well as in vivo. To enhance the effect of fluorescent labeling during the visualization of target loci in the dCas9-GFP system, guide RNAs with aptamers that can attract specific RNA-binding proteins labeled with fluorescent proteins can be used. Compared to techniques such as fluorescent in situ hybridization (FISH), CRISPR imaging offers a unique method for determining chromatin dynamics in living cells [132].

The CRISPR imaging system can be used to dynamically track repetitive and non-repeated genomic loci, as well as to stain chromosomes in living cells. Visualization of a specific genomic locus requires attracting multiple copies of labeled proteins to the selected region. For example, chromosome-specific repeated loci can be efficiently visualized in living cells using a single guide RNA that has multiple target sequences in close proximity. Whereas a non-repetitive genomic locus can be tagged by the simultaneous delivery of multiple guide RNAs that “overlap” the target locus completely. Chromosome staining requires the delivery of hundreds of guide RNAs with target sites distributed throughout the chromosome [133,134,135,136].

CRISPR imaging can be multicolored and allows simultaneous tracking of multiple genomic loci in live cells and in vivo. One method uses orthologous dCas9s labeled with different fluorescent proteins [137]. Alternatively, guide RNAs containing aptamers specific to orthologous RNA-binding proteins with various fluorescent proteins (CRISPRainbow) can be used [133]. The CRISPR-Sirius technology for imaging genomic loci allows modification of guide RNAs with eight different aptamers, providing better stability and signal amplification for imaging [138].

The Casilio system is another CRISPR/Cas9-based system for multiplexed genome locus imaging. Casilio consists of catalytically inactive Cas9, sgRNA appended with one or more PUF-binding site(s), and Pumilio RNA-binding protein with a conserved Pumilio/FBF (PUF) RNA-binding domain that is programmable to bind a specific 8-mer RNA sequence (PUF-binding site). The PUF domains of the Casilio system can be easily programmed to recognize any 8-mer RNA motifs, so this greatly expands the potential number of independent Casilio modules that can be simultaneously delivered into a cell, and each can operate at their defined target sites with independent function. What is more, extensive multimerization of PUF fusions on sgRNA containing PUF-binding site leads to a localized concentration of effectors or protein tags (i.e., fluorescent proteins), which is beneficial for fluorescent imaging [139].

The main advantage of CRISPR/Cas9-based imaging systems is that they can easily be re-targeted to genomic loci of interest. CRISPR/Cas9-based imaging systems may become a powerful tool for studying gene function and chromosome structure, significantly improving the capacity to study the conformation and dynamics of native chromosomes in living human cells [134,139].

### 5.6. dCas9-Based Methods for Isolation of Genomic Loci

The identification of molecules associated with a region of interest in the genome in vivo is important for understanding the functions of the locus. Using dCas9, researchers have improved chromatin immunoprecipitation (ChIP) technology to allow purification of any genomic sequence targeted by guide RNA [140,141,142].

The enChIP technology (engineered DNA-binding molecule-mediated chromatin immunoprecipitation) involves the use of catalytically inactive Cas9 to purify genomic DNA associated with a guide RNA. The epitope tag(s) used for isolation may be fused either with dCas9 or with the guide RNA. Various epitopes, including 3×FLAG, PA, and biotin tags, can be used for enChIP. In addition, antibodies specific for Cas9 can be used to isolate target genomic regions with dCas9. The dCas9-bound locus is isolated by affinity purification according to the epitope used [140,141,142,143,144,145].

Once a target genomic locus has been isolated, all molecules associated with it can be identified using mass spectrometry (used for identification of proteins), RNA sequencing (used for identification of RNAs), and next-generation sequencing (used for identification of DNAs) [142,146,147,148].

Compared to conventional methods used to isolate target genomic loci, CRISPR-based purification methods are simpler and allow direct identification of molecules associated with a genomic region of interest in vivo.

## 6. Base Editors

Two classes of DNA base editors–cytosine base editors (CBEs) and adenine base editors (ABEs)–can be used to introduce single-base changes to DNA without introducing double-strand breaks [42,43,44,149,150,151,152].

CBEs are created by combining Cas9 nickase or catalytically inactive Cas9 with a cytidine deaminase, such as APOBEC (apolipoprotein B mRNA editing enzyme, catalytic polypeptide). CBEs target the desired DNA locus using a guide RNA and can convert cytidine to uridine in a short distance near the PAM site. Subsequently, uridine is repaired to thymidine by the base excision repair mechanism, creating a C to T substitution (or G to A in the complementary strand) [45,46,47].

ABEs have been designed to convert adenosine to inosine, which is repaired by the cell to guanosine, creating an A to G substitution (or T to C in the complementary strand). Like cytosine base editors, the TadA (tRNA adenine deaminase) domain is combined with the Cas9 protein to create the adenine base editor [48,49,50,51].

Both types of DNA base editors are available with several Cas9 variants, including improved Cas9 variants and improved effector domain variants [51,153,154,155,156]. The technology has been improved by optimizing the expression of chimeric proteins, including changes in the linker sequence between the Cas9 protein and the deaminase to customize the editing region or by using chimeric proteins that increase product purity, such as a DNA glycosylase inhibitor (UGI) or a bacteriophage-derived protein Mu (MuGAM) [157,158].

Although many base editors are designed to operate within a very narrow range in the immediate vicinity of the PAM, some are capable of generating a wide range of single nucleotide variants through the process of somatic hypermutation and over a wider editing range, and therefore can be used for directed evolution [159].

Base editors, first reported in 2016 [43], are capable of efficient HIV-1 co-receptor disruption in human T cells and hematopoietic stem progenitor cells [160]. What is more, base editors offer an opportunity for spinal muscular atrophy genetic treatment development [161,162] and for atherosclerotic cardiovascular disease treatment development [163]. Four clinical trials of therapeutics based on CRISPR/Cas9 base editors are mentioned on the CRISPR Medicine News website (https://crisprmedicinenews.com/clinical-trials/ accessed on 5 September 2023): NCT05442346, NCT05456880, NCT05397184, and NCT05398029.

NCT05442346 is a safety and efficacy evaluation study of γ-globin-reactivated autologous hematopoietic stem cells manufactured using glycosylase base editors [164] for the treatment of Thalassemia Major. The estimated study start date was 25 December 2023, but the recruitment was suspended due to the sponsors’ decision (https://classic.clinicaltrials.gov/ct2/show/NCT05442346, accessed on 5 September 2023).

NCT05456880 is a safety and efficacy evaluation of a single dose of autologous CD34+ base-edited hematopoietic stem cells (BEAM-101) to increase fetal hemoglobin (HbF) production in patients with severe sickle cell disease (https://classic.clinicaltrials.gov/ct2/show/NCT05456880, accessed on 5 September 2023). BEAM-101 (Beam Therapeutics Inc., Cambridge, MA, USA) is manufactured using patient-derived hematopoietic stem cells (HSC). HSCs are modified with specific gRNA and base-editor mRNA delivered by electroporation. In preclinical studies, BEAM-101 has shown high levels of HSC editing (over 90% of alleles edited), high and consistent levels of upregulation of HbF (over 60% of total hemoglobin), and significant reductions in the disease-causing protein HbS (less than 40% of total hemoglobin)—levels that are similar to those of sickle cell trait carriers who do not have sickle cell disease [165,166].

NCT05397184 is a phase 1 study of base-editing CAR7 T cells to treat T cell malignancies. The actual study start date is 19 April 2022, and the estimated study completion date is 28 February 2025 (https://classic.clinicaltrials.gov/ct2/show/NCT05397184, accessed on 5 September 2023).

NCT05398029 is an open-label, Phase 1b, single-ascending dose and optional re-dosing study to evaluate the safety of VERVE-101 administered to patients with heterozygous familial hypercholesterolemia, atherosclerotic cardiovascular disease, and uncontrolled hypercholesterolemia (https://classic.clinicaltrials.gov/ct2/show/NCT05398029, accessed on 5 September 2023). VERVE-101 consists of an adenine base editor mRNA (licensed from Beam Therapeutics Inc.) and an optimized guide RNA targeting the *PCSK9* gene packaged with engineered lipid nanoparticles. Inactivation of the *PCSK9* gene has been shown to up-regulate LDL receptor expression, which leads to lower LDL-C levels, thereby reducing the risk of a life-threatening genetic subtype of atherosclerotic cardiovascular disease [167].

Despite their youth, base editors are widely used by both academic laboratories and therapeutic-based companies. Of course, base editors have their limitations, but improvements were made to overcome them. Base editors are already used as therapeutics, and their potential has not yet been exhausted.

## 7. Prime Editors

In October 2019, Andrew Anzalone et al. presented a new genome editing technology called prime editing [52]. Prime editing is a method that allows one to accurately introduce small targeted insertions into the edited DNA sequence to remove and replace bases. Prime editing allows for the introduction of changes into DNA without double-strand breaks. Insertion of target sequences is achieved through the use of donor DNA templates. In addition, prime editing expands the limited range of current DNA base editor capabilities [54]. Instead of Cas9, this method uses the Cas9 nickase combined with reverse transcriptase [52,168].

Currently, there are three main variants of chimeric proteins used for prime editing. The first version of the chimeric protein had low editing efficiency; the second chimeric protein was thermostable and contained additional modifications that led to improved binding to target DNA. Recent versions of chimeric proteins for prime editing have the ability to correct errors that arise during the editing process [52].

The guide RNA used in the prime editing (also known as the pegRNA) is significantly larger than the standard, typically used guide RNAs. This RNA is a guide RNA containing a primer binding site (PBS) and a donor template with the desired sequence added at the 3′ end of the RNA [52]. Currently, such guide RNAs are produced using plasmid DNA and the in vitro transcription method.

During prime editing, a complex of chimeric Cas9 nickase and guide RNA binds to the target DNA and forms a single-strand break. After this, PBS, homologous to the target DNA and located on the guide RNA, binds to a fragment of the target DNA while the donor RNA template is reverse transcribed (reverse transcriptase is a part of the chimeric protein for prime editing) [52]. The target DNA is repaired by new reverse-transcribed DNA, while the original DNA segment is removed by a cellular endonuclease. As a result, one DNA strand is edited.

Third-generation chimeric proteins can correct the unedited DNA strand in the presence of an additional standard guide RNA. In this case, the Cas9 nickase introduces a break into the unedited DNA strand, which is then repaired using the edited DNA strand as a template for nick restoration, thereby completing the editing process [52].

Many different strategies were used for improvement of the prime editing system, including optimization of pegRNA, optimization of the effector proteins, collaborative optimization of prime editors with multiple strategies, an optimization strategy based on inhibiting DNA mismatch repair, and so on [169,170,171,172,173]. In 2022, a novel prime editor was published—Cas9 ortholog-based prime editor. FnCas9(H969A)-RT showed a precise, expanded range of prime editing and versatile editing properties [174].

Prime editing was successfully applied to microorganisms, animal cells, embryos, plants, and human cells [169]. Prime editing expands the genome editing toolkit and can be used for therapeutic development. At the very beginning, prime editing was used to model and then correct sickle cell disease and Tay-Sachs disease [52].

As with all new technological advances, more research is needed to optimize the technology of prime editing. Moreover, it is important to determine whether prime editing can be used in different cell types (especially therapeutically relevant cell types such as primary and stem cells). Also, the long-term effects (if any) and the number of off-target effects of editing need to be investigated.

## 8. Cas-CLOVER

Cas-CLOVER is an RNA-guided endonuclease consisting of the nuclease domain from a *Clostridium* Clo051 type IIs restriction endonuclease fused with catalytically inactive Cas9. Cas-CLOVER activity is based on dimerization of the Clo051 nuclease domain, enabled by RNA-guided recognition of two adjacent 20-nt target sequences. Monomeric Cas-CLOVER does not introduce a nick or a double-strand break. Cas-CLOVER is a high-fidelity site-specific nuclease with low off-target activity that is efficient for genome editing in resting T cells [55], bananas [56] and so on. Cas-CLOVER is also suitable for cell line development [57].

Cas-CLOVER is a proprietary gene editing platform owned by Poseida Therapeutics, Inc. Two clinical trials are mentioned on the CRISPR Medicine News website (https://crisprmedicinenews.com/clinical-trials/, accessed on 5 September 2023).

The first clinical trial (NCT05239143, https://classic.clinicaltrials.gov/ct2/show/NCT05239143, accessed on 5 September 2023) is a phase 1, open label, dose escalation, and expanded cohort study of Allogeneic CAR-T Cells developed using the Cas-CLOVER platform (P-MUC1C-ALLO1) in adult subjects with advanced or metastatic epithelial-derived solid tumors, such as Breast Cancer, Ovarian Cancer, Non-Small Cell Lung Cancer, Colorectal Cancer, Pancreatic Cancer, Renal Cell Carcinoma, Nasopharyngeal Cancer, Head and Neck Squamous Cell Carcinoma and Gastric Cancer. P-MUC1C-ALLO1 targets the MUC1-C epitope and is manufactured using piggyBac^®^ DNA Delivery System, which results in a highly enriched T stem cell memory (T_SCM_) product. It contains 3 transgenes: an anti-MUC1-C humanized scFv-based CAR, a DHFR drug selection gene to improve product homogeneity, and an iCasp9-based safety switch gene to allow for rapid ablation of the CAR-T if required. Cas-CLOVER™ Site-Specific Gene Editing System is used to eliminate expression of endogenous T cell receptors in all cells via knockout of the T cell receptor beta chain 1 gene and expression of MHC class I to prevent and attenuate the graft-versus-host (GvH) response [175].

The second trial (NCT04960579, https://clinicaltrials.gov/study/NCT04960579, accessed on 5 September 2023) is a phase 1, open-label, dose escalation, and multiple cohorts’ study of allogeneic T stem cell memory (T_SCM_) CAR-T cells in subjects with relapsed/refractory Multiple Myeloma. P-BCMA-ALLO1 targets B-cell Maturation Antigen (BCMA) and is manufactured the same way as P-MUC1C-ALLO1 [176].

Among other genome editing technologies, Cas-CLOVER is a relatively young technology—the first time it was mentioned on 14 April 2020. To date, this high-precision gene editing technology has been successfully validated in mammalian cells as a genome editing tool with zero detected off-target activity. Two clinical trials of allogeneic CAR-T cells developed using the Cas-CLOVER platform were initiated in 2021 and 2022.

## 9. Cas-FokI

Cas-FokI is a dimeric RNA-guided FokI nuclease, also known as RFNs (RNA-guided FokI Nucleases), that can recognize extended sequences and edit genes with high efficiencies in human cells. Cas-FokI target size is about 38–40 bp plus spacer length (i.e., length between two targets, 16–18 bp or 26 bp, depending on variant used) [61]. Like Cas-CLOVER, the activity of Cas-FokI is based on dimerization of the FokI nuclease domain, which depends strictly on the binding of two guide RNAs to DNA [41]. RFN monomers can also introduce undesired point mutations at their target sites, although at a frequency lower than that for Cas9 nickases [41]. Point mutation rates induced by Cas9 nickases are up to 10-fold higher than the point mutation rates of monomeric RFNs [58].

Cas-FokI provides a versatile genome-editing tool with improved specificity and catalytic activity for precise engineering of mammalian genomes [59,60,61]. For example, RFNs were successfully used in multiple studies with model animals [61] and cell-line models (Phenylketonuria [177] and chronic myeloid leukemia [62]).

Cas9-derived proteins, e.g., base editors, prime editors, Cas-CLOVER, and Cas-FokI differ from regular SpCas9 (see Table 3 below) and allow (i) to introduce single base changes to DNA, (ii) to introduce small targeted insertions into DNA, remove and replace bases, and (iii) precisely edit DNA with undetectable off-target effects. All the editing tools mentioned are popular among scientists to varying degrees and can be used for therapeutic approach development in the near future.

## 10. CRISPR/Cas9 Delivery Methods

The success of genome editing depends on the specificity and efficiency of the Cas9 protein, the design of the guide RNA, and the efficiency of the delivery of elements of the CRISPR/Cas system into the target cell. CRISPR/Cas9 elements can be delivered using different methods, i.e., physical methods, viral and non-viral vector delivery, etc. Physical methods of delivery imply short-term disruption of the target cell membrane and include electroporation, sonoporation, nano-injection, micro-injection, and hydrodynamic injection [178]. Viral vectors are the earliest molecular tools for gene transfer to human cells; they transfer nucleic acids encoding CRISPR/Cas9 components to target cells in the envelope of a virus, for example, an adenovirus, adeno-associated virus, retrovirus, lentivirus, Epstein–Barr virus, herpes simplex virus, and bacteriophages [179,180]. In addition, alternative (non-viral) methods of CRISPR/Cas9 delivery, for example, by using lipid nanoparticles, polymer and hydrogel nanoparticles, hybrid gold, graphene oxide, metal-organic frameworks, black phosphorus nanomaterials, etc., were reported [181].

Cas9 and guide RNA can be delivered to a cell via three different modes: (i) as a set of plasmid DNAs; (ii) as a combination of Cas9 mRNA and guide RNA; and (iii) as pre-assembled ribonucleoprotein complexes (RNPs) (Table 4).

**Figure 3:**
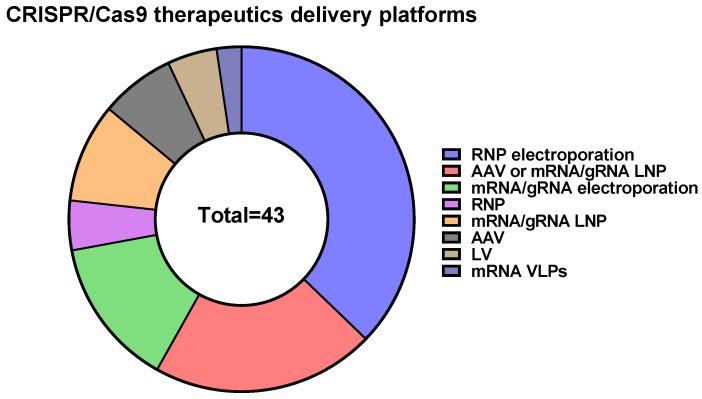
Delivery approaches for CRISPR/Cas9 therapeutics in clinical trials are mentioned on the CRISPR Medicine News website (https://crisprmedicinenews.com/clinical-trials/, accessed on 5 September 2023).

To date, many strategies are available for CRISPR/Cas9 RNP delivery based on physical approaches and synthetic carriers. CRISPR/Cas9 RNPs were successfully delivered to target cells using microinjection [182], biolistics [183,184], electroporation [185,186,187,188,189], microfluidics [190,191], filtroporation [192], nanotube [193], osmocytosis [194], synthetic lipid nanoparticles [195], cell penetrating peptides (CPPs) [196], lipopeptides [197], dendrimers [198], chitosan nanoparticles [199], nanogels [200], gold nanoparticles [201], metal-organic frameworks [202], graphene oxide [203], black phosphorus nanosheets [204], calcium phosphate nanoparticles [205], and many more [206]. It should be mentioned that CRISPR/Cas9-based therapeutics predominantly use electroporation of RNPs into target cells as a delivery method (Figure 3).

Delivery of CRISPR/Cas in the form of RNPs is believed to have several advantages, including high editing efficiency, low nonspecific activity, editing beginning immediately after delivery to the cell, the ability to quickly screen the effectiveness of guide RNAs in vitro, and decreased immunogenicity due to the transient presence of CRISPR/Cas elements in the target cell. Thus, RNPs offer promising opportunities for CRISPR/Cas9-based genome editing. However, RNP delivery is rather difficult due to the high molecular weight of Cas9 protein (~160 kDa) and the additional requirement for optimization of RNP loading with sgRNA. Moreover, several limitations are associated with RNP delivery due to the charge properties of the complex. There are a lot of methods for RNP delivery, but some of them seem to be expensive and cannot be scaled up; others carry an intellectual property burden. More investigations and optimizations are required to overcome the existing problems of Cas9 RNP delivery.

## 11. CRISPR/Cas9-Based Diagnostics

Since 2017, CRISPR/Cas proteins with collateral cleavage activity–Cas12 and Cas13, have been used in the field of molecular diagnostics [207,208,209,210]. The main feature of such diagnostics is that CRISPR/Cas complexes “recognize” target sequences with excellent specificity and subsequently cleave labeled reporter molecules. The Cas9 protein, the best-known representative of the Cas protein family, is also used for diagnostics, but the mode of action is different: the labeled CRISPR/Cas complexes “recognize” target sequences.

In 2016, CRISPR/Cas9 was for the first time mentioned as a part of a paper-based sensor to detect clinically relevant concentrations of Zika virus and to discriminate between closely related viral strains with single-base resolution. The assay was called NASBACC (from NASBA, Nucleic Acid Sequence-Based Amplification, and CRISPR Cleavage), had a colorimetric readout, and an LOD (limit of detection) = 6 × 10^5^ copies/ml [211]. Also, Vilhelm Müller et al. described an analysis based on optical DNA mapping of individual plasmids carrying antibiotic resistance genes of bacterial isolates in nanofluidic channels, which provides detailed information about these plasmids, including the presence/absence of antibiotic resistance genes. The described assay allowed the identification of antibiotic resistance genes using CRISPR/Cas9 and antibiotic resistance gene-specific guide RNAs (blaCTX-M group 1, blaCTX-M group 9, blaNDM, and blaKPC). During the analysis, the CRISPR/Cas9 ribonucleoprotein complex linearizes circular plasmids in the region of the antibiotic resistance gene, and the resulting linear DNA molecules are identified using optical DNA mapping [212].

Later, CRISDA [213], CAS-EXPAR [214], CRISPR-Chip [215], and FLASH-NGS [216] technologies were developed. CRISDA combines the strand-displacement amplification technique with the Cas9-mediated target enrichment approach and exhibits sub-attomolar sensitivity with an LOD = 1.5 × 10^2^ copies/ml [213]. The CAS-EXPAR technique has comparable sensitivity (LOD = 4.9 × 10^2^ copies/ml) and does not require exogenous primers (primers are first generated by Cas9/sgRNA-directed site-specific cleavage of the target and accumulated during the reaction) [214]. CRISPR-Chip represents ribonucleoprotein complexes formed by catalytically inactive Cas9 and target-specific sgRNAs immobilized on the surface of the graphene layer. When RNPs bind target DNAs, it causes a change in electrical current, which makes for a simple signal readout. CRISPR-Chip allows detection of femtomolar amounts of DNA without the need for target preamplification [215]. In 2019, FLASH-NGS was introduced as a unique technology for sub-attomolar detection of low-abundance pathogen sequences. In FLASH-NGS, Cas9 is used to enrich (up to 5 orders of magnitude) the sample with a programmed set of sequences [216].

SARS-CoV-2 pandemic gave rise to CRISPR/Cas9-based methods named FELUDA [217], CASLFA [218], VIGILANT [219], “Biotin-dCas9-LFA” [220], LEOPARD [221], and Bio-SCAN [222].

FELUDA (FnCas9 Editor-Linked Uniform Detection Assay) is a semi-quantitative assay utilizing direct catalytically inactive FnCas9-based detection of PCR-amplified sequences. Target sequence in FELUDA is labeled with biotin and RNP–with FAM/FITC, which allows detection of results with lateral flow readout. FELUDA can detect nucleic acids with high sensitivity/specificity, and its LOD is 10 copies per reaction [217].

CASLFA (Cas9-mediated lateral flow nucleic acid assay) can be performed in two different ways. The first option includes biotinylated amplicons, target-specific Cas9/sgRNA complexes, and AuNP-DNA probes, which are hybridized with the single-strand region of the amplicon released by Cas9/sgRNA-mediated unwinding. The second option includes sgRNA with a universal sequence in the stem-loop region for AuNP-DNA probe hybridization, biotinylated amplicon, and a target-specific Cas9/sgRNA complex. CASLFA can detect 200 copies per reaction [218].

VIGILANT (VirD2-dCas9-guided and LFA-coupled nucleic acid test) is a nucleic acid detection technology based on the use of a fusion of catalytically inactive SpyCas9 and VirD2 relaxase. Target sequence is amplified using biotinylated oligos and is specifically bound by dCas9, while VirD2 covalently binds to a FAM-tagged oligonucleotide. Afterwards, the biotin label and FAM tag are detected by any available LFA with a limit of detection of 2.5 copies/μL [219].

The Biotin-dCas9-LFA assay includes FAM-labelled amplicon, biotinylated target-specific dCas9/sgRNA complex (bdCas9), and a competing PAM-rich soak double-stranded oligonucleotide to prevent non-specific bdCas9/mismatched sgRNA binding. It should be noted that the biotin-dCas9-LFA limit of detection (LOD) is similar to that of qRT-PCR [220].

Chunlei Jiao et al. found that RNA guides from Cas9-RNA complexes from *Campylobacter jejuni* can also originate from cellular RNAs unassociated with viral defense. This fact led to the reprogramming of tracrRNAs so that they could link the presence of any RNA of interest to DNA targeting with different Cas9 orthologs (CjeCas9, SpyCas9, and Sth1Cas9). This work gave rise to a multiplexable, ultrasensitive diagnostic platform named LEOPARD (leveraging engineered tracrRNAs and on-target DNAs for parallel RNA detection) [221].

Bio-SCAN (biotin-coupled specific CRISPR-based assay for nucleic acid detection) is a simple, rapid, specific, and sensitive pathogen detection platform that does not require sophisticated equipment or technical expertise. Within 1 h of sample collection, Bio-SCAN can detect a clinically relevant level (4 copies/μL) of the SARS-CoV-2 RNA genome. Bio-SCAN consists of FAM-tagged oligonucleotides, biotinylated catalytically inactive SpyCas9, and AuNP-anti-FAM antibodies. The target nucleic acid sequence is amplified in 15 min via RPA and then detected on commercially available lateral flow strips [222].

CRISPR/Cas9 offers an opportunity for the development of a wide variety of point-of-care diagnostics (more than 10 diagnostic platforms have been published to date), but it still remains an investigational rather than practical approach. CRISPR/Cas9-based pathogen detection platforms are rather simple, possess excellent specificity, are very sensitive (and even ultrasensitive), and are able to detect clinically relevant levels of pathogen-specific nucleic acids. Some CRISPR/Cas9-based nucleic acid detection platforms may become the basis for express point-of-care laboratory and home tests.

## 12. CRISPR/Cas9-Based Therapeutics

Nowadays, CRISPR/Cas is used to study and develop therapeutic approaches for the treatment of a wide variety of human diseases [2,223,224,225,226,227].

Genome editing using CRISPR/Cas systems is used to develop antiviral therapy to treat infectious diseases. The therapeutic effect is to be achieved either by altering host genes important for the viral life cycle or by targeting viral genes required for replication [228]. Today, several approaches to HIV therapy development are based on genome editing technology.

CRISPR/Cas9 has been used to induce site-specific genome modification in human cells in vitro and in vivo using mouse models of HIV infection [229,230,231,232,233]. Numerous academic laboratories have successfully performed CD4+ T cell CCR5 receptor knockouts using CRISPR/Cas9. This approach was shown to inhibit HIV-1 infection without significant side effects [232]. CCR5 editing in both the hematopoietic stem cell (HSC) population and the CD4+ T lymphocyte population is a promising strategy for creating HIV-resistant cells. However, this approach is ineffective against CXCR4 (C-X-C chemokine receptor type 4)-tropic HIV strains. It has been shown that CRISPR/Cas9 can be used to edit the gene encoding CXCR4 with high precision and efficiency. Knockout of the HIV co-receptor CXCR4 is accompanied by minor off-target effects and provides resistance to HIV infection caused by CXCR4-tropic HIV strains [230,234,235,236]. This approach could be used to generate experimental and therapeutic primary human CD4+ T cells, providing an alternative treatment for HIV-1 X4 infection. At the same time, simultaneous knockout of both HIV co-receptors Chemokine C-C-Motif Receptor 5 (CCR5) and CXCR4 leads to a decrease in the expression of CCR5 and CXCR4, which makes the modified cells resistant to infection with R5 and X4 tropic viruses, even when using double tropic viruses [230].

In recent years, CRISPR technology has been successfully used to reduce or eliminate persistent viral infections in vitro and in animal models in vivo, raising the prospect of its application in the treatment of latent and chronic viral infections [237].

CRISPR/Cas technologies have been used to combat HIV infection in vitro in various cell lines. At the same time, it was possible to achieve not only suppression of HIV gene expression in infected T cells and microglial cells but also to remove HIV proviral DNA from many other cell lines, including neuronal progenitor cells, which represent latent reservoirs of HIV infection [238,239,240]. CRISPR/Cas systems have also been shown to be effective in combating HIV infection in vivo. Thus, HIV proviral DNA was eliminated from the spleen, lungs, heart, colon, and brain of animals in a humanized model of chronic HIV infection [240]. In addition, using CRISPR/Cas technology, HIV proviral DNA was removed from infected human peripheral blood mononuclear cells using a transgenic mouse model [241].

In 2017, the CRISPR/Cas9 system was used to remove a full-length fragment of hepatitis B virus (HBV) DNA that was chromosomally integrated and episomally localized as cccDNA in chronically infected cells. This approach allowed complete eradication of HBV in a stable infected cell line in vitro. This suggests that the CRISPR/Cas9 system is a potentially powerful tool for eradicating chronic HBV infection and curing HBV completely [242,243].

In addition, the CRISPR/Cas system has been successfully used to combat herpesvirus infections in vitro. It was shown that the simultaneous use of several guide RNAs made it possible to significantly reduce the replication of herpes simplex virus 1 in cells [244,245]. Using CRISPR/Cas, it was also possible to eliminate up to 95% of the DNA of the Epstein-Barr virus and cytomegalovirus within 11 days, after which mutant forms of the virus appeared, resistant to the action of CRISPR/Cas [244]. CRISPR/Cas systems have also been shown to eliminate other viral pathogens in vitro, such as the John Cunningham virus and the human papillomavirus HPV-16 and HPV-18 [246,247].

CRISPR/Cas9 was used to develop therapeutic approaches for the treatment of monogenic diseases such as cystic fibrosis [248,249,250,251], sickle cell disease [30,252,253,254], thalassemia [255,256,257,258], Huntington’s disease [259,260,261,262,263], Duchenne muscular dystrophy [264,265,266,267,268,269,270], hemophilia [271,272,273,274,275], diabetes [276,277,278,279,280] and cardiovascular diseases [281,282,283,284,285].

What is more, novel therapeutic approaches for cancer treatment are based on CRISPR/Cas9. CRISPR/Cas9 was used for the development of CAR-T cells (T cells with a chimeric antigen receptor) that have high antitumor activity, including “universal” CAR-T allogeneic T cells on which endogenous T-cell receptor (TCR) and Human Leukocyte Antigen (HLA) are eliminated [286,287,288,289,290,291,292,293,294,295,296,297,298]. Also, CRISPR/Cas9 was used to produce CAR-T cells in which a CAR or TCR cassette was introduced into the endogenous TCR gene locus to mitigate graft-versus-host disease, preventing random integration of the cassettes and ensuring uniform CAR (chimeric antigen receptor) expression [299,300,301,302].

**Figure 4:**
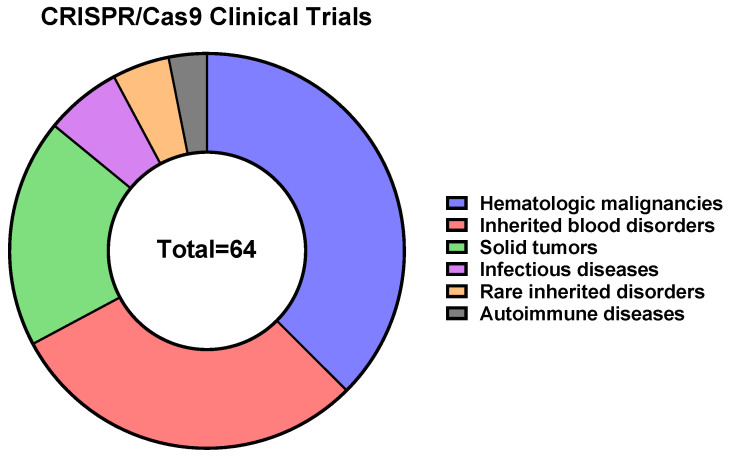
The number of CRISPR/Cas9 clinical trials mentioned on the CRISPR Medicine News website (https://crisprmedicinenews.com/clinical-trials/, accessed on 5 September 2023).

To date, 64 CRISPR/Cas9-, 4 CRISPR/Cas9 base editors-, and 2 Cas-CLOVER-based therapeutics are in clinical trials, according to the CRISPR Medicine News website (https://crisprmedicinenews.com/clinical-trials/, accessed on 5 September 2023). The vast majority of CRISPR/Cas9-based therapeutics are directed against hematologic malignancies (~38%), inherited blood disorders (~29%), and solid tumors (~19%) (Figure 4, Supplementary Table S1).

Recently, encouraging news has emerged: some patients are functionally cured of sickle cell disease or beta thalassemia, and the edited cells reside in the bone marrow, indicating the potential for long-term treatment. Cancer immunotherapy trials are in the early stages, but the safety and tolerability of the treatments look promising moving forward with newer versions of the editing technology, off-the-shelf products, moving toward new cancer targets, and even developing new cell types for immunotherapy.

All treatment methods mentioned above are relatively new. Positive results still require long-term follow-up to see whether the treatment remains effective, whether patients suffer unwanted changes, and whether patients have immune responses against Cas proteins.

## 13. Pre-Existing Immunity against CRISPR/Cas9 Proteins

The most widely used Cas9 orthologs are derived from *Staphylococcus aureus* (SaCas9) and *Streptococcus pyogenes* (SpyCas9) [303], which are common human commensals. There is still no accurate data on pre-existing antibodies (i.e., humoral immunity) against SaCas9 and SpCas9. The prevalence of anti-SaCas9 antibodies ranges from 4.8% to 100% (10% [304], 78% [305], 95% [306], 4.8% [307], and 100% [308]). At the same time, the prevalence of anti-SpCas9 antibodies ranges from 0% to 100% (2.5% [304], 58% [305], 5% [227], 0% [296], 95% [306], and 100% [308]).

Data on pre-existing cellular immunity against SaCas9 and SpCas9 have a much smaller spread of values. Namely, pre-existing cellular immunity against *Staphylococcus aureus* Cas9—78% [305], 100% [309], 88–96% [306], and 70% [307]. While pre-existing cellular immunity against *Streptococcus pyogenes* Cas9—67% [305], 95% [309], 83% [227], 66.7% [296], and 92–96% [306].

To overcome the pre-existing humoral immunity, Cas9 proteins were subjected to protein engineering. It was shown that the SaCas9 variant bearing an R338G substitution reduces B cell immunogenicity and retains its gene-editing function [307]. Also, engineering Cas9 epitopes recognized by circulating cytotoxic CD8+ T cells can diminish pre-existing cellular immunity [227]. Alternatively, Cas systems isolated from bacteria to which humans have not been exposed can be used to solve the problem of pre-existing immunity [310]. If necessary, multiple immunosuppressive drug treatments, including T cell-depleting regimens (approved for AAV-based therapies), are available [311,312,313,314,315]. However, some data indicate that pre-existing immunity does not impair the engraftment of CRISPR/Cas9-edited cells in pre-conditioned busulfan or radiation organisms [316].

Pre-existing humoral and cell-mediated adaptive immune responses to Cas9 in humans should be taken into account when CRISPR/Cas9-based therapeutics enter clinical trials.

## 14. CRISPR/Cas9 Activity Modulators

CRISPR/Cas systems are bacterial anti-viral systems, and bacterial viruses (bacteriophages, phages) can carry anti-CRISPR (Acr) proteins to evade that immunity. Acrs can also fine-tune the activity of CRISPR-based genome-editing tools.

More than 50 verified Acrs (~90% anti-CRISPR Cas9 type II-A, ~10% anti-CRISPR Cas9 type II-C) are mentioned in the Anti-CRISPRdb database (http://guolab.whu.edu.cn/anti-CRISPRdb/, accessed on 5 September 2023, Supplementary Table S2) [317,318]. These Acrs belong to the AcrIIC1-AcrIIC5, AcrIIA1-AcrIIA22, and AcrIIA24-AcrIIA32 families and can inhibit NmeCas9 (*Neisseria meningitidis*), SpyCas9 (*Streptococcus pyogenes*), HpaCas9 (*Haemophilus parainfluenzae*), SmuCas9 (*Simonsiella muelleri*), St1Cas9 (*Streptococcus thermophilus*), SauCas9 (*Staphylococcus aureus*), SinCas9 (*Streptococcus iniae*), and St3Cas9 (*Streptococcus thermophilus*) to different extents [73,74,75,319,320,321,322,323,324,325,326,327].

The use of Acrs became a proven method for minimizing the off-target effects of CRISPR/Cas tools in various hosts [328,329,330]. Artificially weakened anti-CRISPR (Acr) proteins can be used to reduce CRISPR/Cas off-target effects. If Acr is co-expressed with or directly fused to Cas9, it can fine-tune Cas9 activity toward selected levels to achieve an effective kinetic insulation of on- and off-target editing events [331]. Moreover, several chemically inducible Acrs were developed by comprising hybrids of Acr protein and the 4-hydroxytamoxifen-responsive intein. Such systems enabled post-translational control of CRISPR/Cas9-mediated genome editing in human cells [327].

In addition to Acrs, small molecules are capable of modulating the activity of CRISPR/Cas9 systems. Small molecules are used to arrest cells at G1/S, G2/M, or S phases, regulate chromatin accessibility, inhibit NHEJ-mediated repair, and promote HDR [332].

After additional investigations, anti-CRISPR proteins and small molecules may become promising ways for precisely controlling and enhancing CRISPR/Cas9 genome editing efficacy and specificity.

## 15. Cas9 Orthologs

Cas9 proteins are relatively large, with an average length of 1252 ± 165.8 aa (mean ± SD, range 961–1809 aa). More than 10,000 Cas9 orthologs are present among *Bacillota, Bacteroidota, Pseudomonadota*, and other phylums. Cas9 is most frequently found in microorganisms belonging to the *Streptococcus, Listeria, Staphylococcus, Bacteroides*, and *Neisseria* genera (Supplementary Table S3).

All Cas9 proteins can be roughly divided into two types: common-sized Cas9 proteins and miniature Cas9 proteins. Miniature Cas9 proteins are more advantageous in terms of delivery and belong to the *Staphylococcaceae, Akkermansiaceae, Lactobacillaceae, Sphingomonadaceae, Corynebacteriaceae, Neisseriaceae, Bacillaceae, Pasteurellaceae, Comamonadaceae, Helicobacteraceae*, and *Campylobacteraceae* families (Supplementary Table S4).

Many Cas9 orthologs (e.g., ScCas9 [177], CjCas9 [333], NmeCas9 [334], FnCas9 [335], SmacCas9 [336], SauriCas9 [337], St1Cas9 [338], St3Cas9 [339], FrCas9 [76], and so on [340,341,342,343,344,345,346,347,348,349,350,351,352,353,354,355,356,357,358]) have been reported to be intrinsically high-fidelity [76,77], and some of them were engineered with a broad PAM range, high fidelity, and/or both [359] (Supplementary Table S4).

CRISPR/Cas9 orthologs significantly expand the range of available molecular tools for genome editing and can be chosen when it is important to overcome the problem of pre-existing immunity and/or anti-CRISPR (Acr) proteins. Moreover, CRISPR/Cas9 orthologs can be used for proprietary genome editing approaches to overcome the burden of intellectual property on the widely used CRISPR/Cas9 variants. However, additional studies are needed to evaluate the off-target effects of the selected CRISPR/Cas9 orthologs.

## 16. Future Perspectives

If the diversity of the CRISPR/Cas9 toolbox is not enough, the researcher can easily turn to other CRISPR/Cas proteins, such as Cas12, Cas13, and Cas14.

The gene, originally designated *cpf*1, is present in a number of bacterial and archaeal genomes, where it coexists with the *cas*1 and *cas*2 genes and the CRISPR array [360]. Cas12a (Cpf1) is the prototype effector of type V multidomain Cas proteins. Cpf1 contains two RuvC-like nuclease domains but lacks an HNH domain. Structural analysis of the Cas12a-crRNA target DNA complex revealed a second nuclease domain (Nuc) with a unique structure, functionally similar to the HNH domain of Cas9 [361]. Cas12a is an RNA-dependent DNA endonuclease that does not require tracrRNA, which is essential for Cas9 activity [135]. The protein also differs from Cas9 in its cleavage pattern and PAM range. More than 30 Cas12 proteins are well characterized [362,363,364,365,366,367,368]. For example, AsCas12a (from *Acidaminococcus* sp.) is a differentiated CRISPR nuclease with higher specificity and efficiency compared with Cas9 [369]. Two clinical trials of therapeutics based on CRISPR/Cas12 sponsored by Editas Medicine, Inc. are mentioned on the CRISPR Medicine News website (https://crisprmedicinenews.com/clinical-trials/, accessed on 5 September 2023)–NCT04853576 (Sickle Cell Disease, https://clinicaltrials.gov/study/NCT04853576, accessed on 5 September 2023) and NCT05444894 (Transfusion Dependent Beta-Thalassemia, https://clinicaltrials.gov/study/NCT05444894, accessed on 5 September 2023). All trial participants experienced early successful engraftment with no serious adverse events. The safety profile of CRISPR/Cas12-based treatment was consistent with busulfan myeloablative conditioning and autologous hematopoietic stem cell transplantation. Importantly, no patients experienced vaso-occlusive events following therapy.

The discovery of two distantly related class 2 effector proteins, Cas9 and Cas12a, suggested that other variants of such systems may exist. Indeed, soon among class II effectors, through a targeted search (bioinformatics analysis), the proteins Cas12b (type V), Cas13a, and Cas13b (type VI), which differ from Cas9 and Cas12a, were discovered, and their activity was confirmed [362]. Type V effectors, such as Cas9, use tracrRNA for targeted activity. To date, most functionally characterized CRISPR/Cas systems have been reported to target DNA, and only type IIIA and IIIB multicomponent systems can cleave RNA [370]. Type VI effectors Cas13a and Cas13b specifically target RNA, thereby mediating RNA interference. Unlike type II and type V effectors, Cas13a and Cas13b do not have RuvC-like nuclease domains and instead contain a pair of HEPN domains (higher eukaryotes and prokaryotes nucleotide-binding domains) [371]. More than 49 Cas13 proteins are well characterized [207,372,373,374,375].

In 2018, a novel CRISPR/Cas cassette containing *cas*1, *cas*2, *cas*4, and a new gene, *cas*14, was discovered. Cas14 encodes a miniature Cas protein (molecular weight 40–70 kDa), which is half the size of other Cas proteins found in class 2 CRISPR/Cas systems [376]. There are 24 variants of the cas14 gene, which are grouped into 3 subgroups (*cas*14a-c). All variants contain the predicted RuvC nuclease domain. Unlike other Cas enzymes, Cas14 has not been found in bacterial genomes but only in the genome of the archaeal group. Therefore, it is suggested that Cas14 may be a more primitive version of the larger and more complex proteins Cas9 and Cas12. Cas14 (also known as Cas12f) can bind and cleave the target single-stranded DNA sequence. Unlike Cas9, Cas14 does not require a PAM sequence [376,377,378].

Like Cas12, Fanzor (Fz) proteins are believed to be putative descendants of the recently discovered OMEGA effector TnpB (Transposase B, Transposon-associated protein) [379,380]. Fz proteins are RNA-guided endonucleases with relatively small sizes compared with Cas9/12 [381]. Approximately 649 Fanzor proteins from various eukaryotic species form two distinct large classes: Fz1 and Fz2. Fz1 proteins are found in fungi (in incertae sedis species), but they can also be seen in protists, arthropods, plants, and eukaryotic viruses (giant viruses). Fz2 is spread among fungi, molluscs, choanoflagellates, and eukaryotic giant viruses [381]. Like CRISPR/Cas, Fanzor proteins can be reprogrammed for human genome engineering applications, representing an attractive starting point for further therapy development [381], but further investigations are needed.

The CRISPR/Cas9 toolbox itself is extremely diverse, and at the same time, there is a huge variety of other closely related molecular tools for genome editing that can be used for the same needs as CRISPR/Cas9 (see Table 5).

## 17. Conclusions

CRISPR/Cas9 and its derivatives are unique molecular tools that can be easily used in a wide variety of applications, such as genome engineering via different modes of action (cut, nick, base edit, and prime edit); transcription regulation and screening via CRISPRa and CRISPRi; epigenetic editing and using sgRNA libraries; genome loci of interest visualization, purification, tagging, and so on. CRISPR/Cas9 is used as a diagnostic tool and for the development of therapeutic approaches (more than 60 clinical trials are being conducted at the moment). The CRISPR/Cas9 genome engineering toolbox includes variants with improved efficacy and specificity. More than 100 CRISPR/Cas9 orthologs with known PAM specificity are well characterized and can be used for all the applications mentioned above.

Delivery of CRISPR/Cas9 to target cells still remains the biggest bottleneck for widespread clinical use of CRISPR/Cas9 in human therapy. Many efforts are made to develop efficient CRISPR/Cas delivery methods that will not affect the viability of the target cell dramatically. A lot of methods and protocols for CRISPR/Cas9 delivery exist, and electroporation and viral vectors remain the gold standard to date.

On the one hand, CRISPR/Cas9 is an indispensable molecular tool for studying biological systems, signaling pathways, and their role in the pathogenesis of human diseases. CRISPR genome-wide screening offers great promise for discovering important disease-associated genes and revealing potential therapeutic targets. On the other hand, CRISPR/Cas9 offers an option to overcome genetic diseases in the near future. Its ability to precisely knock out specific genes without large-scale chromosomal rearrangements enables multiple gene knockouts in human cells to generate more efficient and safer cell therapies. The use of CRISPR/Cas9 gave rise to a novel type of immuno-oncology therapies—“off-the-shelf” (allogeneic) universal CAR-T cells, which are widely used in clinical trials.

In summary, CRISPR/Cas9 has had a great impact on many fields of fundamental and applied research and will continue to play an important role in the future.

## Figures and Tables

**Table 1:** CRISPR/Cas9 improved variants.

Name	Key Features	Reference
Cas9 variants engineered using random mutagenesis combined with high-throughput screening		
Sniper-Cas9	Sniper-Cas9 showed much higher on-target activities and comparable specificity at most off-target loci when compared with other engineered high-fidelity SpCas9 variants such as SpCas9-HF1, HypaCas9, evoCas9, and eSpCas9(1.1). However, Sniper-Cas9 showed stronger tolerance to single mismatches in the PAM-distal region (e.g., 16th, 18th, and 19th). Sniper-Cas9 showed on-target activities with extended or truncated sgRNAs and worked well in a preassembled ribonucleoprotein (RNP) format to allow DNA-free genome editing	[33]
HiFi Cas9	HiFi Cas9 (R691A variant) demonstrated the highest on-target activity (82%) in the RNP format compared to other improved Cas9 variants	[30]
xCas9	Cas9 variant with expanded PAM compatibility (xCas9 can recognize a broad range of PAM sequences, including NG, GAA, and GAT). xCas9 demonstrated lower genome-wide off-target activity at all NGG target sites, as well as minimal off-target activity when targeting genomic sites with non-NGG PAMs	[38]
SpartaCas	SpartaCas (D23A, T67L, Y128V, and D1251G variants) had reduced off-target events while maintaining high on-target editing in T-cells. The editing efficiency of SpartaCas was slightly reduced when compared to wild-type Cas9, but it demonstrated dramatically higher editing efficiency when compared to SpCas9-HF4 and eCas9	[34]
efSaCas9	efSaCas9 (N260D variant) is a high-fidelity, high-activity variant that could be harnessed for safe and reliable genome editing. efSaCas9 demonstrated dramatically reduced off-target effects (approximately 2- to 93-fold improvements) compared to wild-type SaCas9	[28]
Cas9 variants engineered using a structure- and/or function-guided protein engineering approach		
SpCas9-D1135E	SpCas9-D1135E possessed genome-wide specificity and demonstrated reduced off-target effects. The D1135E mutant was able to better discriminate between NGG and NGA PAMs compared with wild-type SpCas9. Moreover, it had decreased activity against non-canonical NAG, NGA, and NNGG PAMs relative to wild-type SpCas9	[25]
eSpCas9	eSpCas9 variants, also known as “enhanced specificity”, SpCas9 showed reduced off-target effects and maintained robust on-target cleavage.	[27]
SpCas9-HF1 (and -HF2, -HF3, -HF4)	SpCas9-HF are high-fidelity SpCas9 variants harboring alterations designed to reduce non-specific DNA contacts. SpCas9-HF retained on-target activities comparable to wild-type. SpCas9-HF rendered all or nearly all off-target events undetectable, even for atypical, repetitive target sites	[24]
HypaCas9	HypaCas9 (N692A, M694A, Q695A, and H698A variants) exhibited dramatically improved genome-wide specificity compared to wild-type SpCas9 and showed equivalent or better genome-wide specificity relative to both SpCas9-HF1 and eSpCas9 variants	[31]
SuperFi-Cas9	SuperFi-Cas9 was able to discriminate between on- and off-target substrates without compromising DNA cleavage efficiency, but a recent study showed that it had significantly reduced on-target activity in mammalian cells	[36,84]
LZ3 Cas9	The LZ3 Cas9 variant is known to exhibit high activity, increased specificity, and a differential +1 insertion profile as compared to wild-type SpCas9. Further rational engineering of LZ3 Cas9 might provide novel opportunities for non-templated correction of disease-causing frameshift mutations in the human population	[37]
eSaCas9	eSaCas9 is a high-fidelity version of SaCas9 obtained by weakening interactions between Cas9 and the target DNA. eSaCas9 demonstrated high on-target activities and comparable specificity at most off-target loci	[27]
SaCas9-HF	SaCas9-HF showed high genome-wide targeting accuracy without compromising on-target efficiency, as validated by rigorous evaluation of its on- and off-target activities across multiple endogenous sites	[32]
KKH-SaCas9-SAV1 (and -SAV2)	KKH-SaCas9-SAV1 and SAV2 are SaCas9 variants that exhibited low off-target and high on-target activities and revealed a pivotal role of the previously unreported Y239H substitution in determining target accuracy while maintaining the activity of KKH-SaCas9	[39]
Cas9 variants engineered using directed evolution combined with structure-guided modeling		
evoCas9	EvoCas9 is a variant that has fidelity exceeding both wild-type (79-fold improvement) and rationally designed Cas9 variants (4-fold average improvement), while maintaining near-wild-type on-target editing efficiency (90% median residual activity)	[29]
SaCas9-Q414A	The Q414A variant of SaCas9 exhibited even higher fidelity than the N260D variant of SaCas9 while retaining the most on-target activity	[28]
Cas9-based fusion proteins with improved properties		
miCas9	Cas9 variant with improved homology-directed repairing capacity (2.5-fold higher). To improve Cas9’s homology-directed repair capacity, SpCas9 was fused to a minimal motif consisting of thirty-six amino acids (Brex27 motif)	[40]
Cas9-pDBD	Fusing a programmable DNA-binding domain (pDBD) to Cas9 combined with the attenuation of Cas9’s inherent DNA binding affinity produced a Cas9-pDBD chimera with dramatically improved precision and increased targeting range	[85]
Cas-Cas9 chimeras	RNA-programmable Cas9-Cas9 chimeras, in single- and dual-nuclease formats, were designed as versatile genome engineering systems. In both formats, Cas9-Cas9 fusions displayed an expanded targeting repertoire and achieved highly specific genome editing. Dual-nuclease Cas9-Cas9 chimeras had higher target site activity and generated predictable, precise deletion products between target sites	[86]

**Table 2:** dCas9-fusion proteins for transcriptional and epigenetic regulation.

Action	Name	Function	Reference
Transcription repression	dCas9 (CRISPRi)	Steric hindrance	[92,93,94,95,96]
	dCas9-KRAB, dSaCas9-KRAB	Histone methylation and deacetylation	[90,93,97,98,99,100,101,105]
	dCas9-KRAB-MeCP2	Represses transcription from methylated promoters via binding	[102]
	ZIM3-dCas9	Histone methylation and deacetylation	[95,103]
	dCas9-SALL1-SDS3	Histone deacetylation	[104]
	dCas9-SRDX	Transcriptional repressor domain (in plants)	[106,107,108]
Transcription activation	dCas9-VP64	Transcriptional activator	[109]
	dCas9-SAM	Multiple copies of transcriptional activators	[109]
	dCas9-SunTag	Multiple copies of transcriptional activators	[111,112]
	dCas9-SPH	Multiple copies of transcriptional activators	[113]
	dCas9-VPR	Combination of three transcriptional activators	[114,115]
Epigenetic repression	dCas9-LSD1	Histone demethylation	[123,124]
	dCas9-DNMT3A	DNA methylation	[128]
	dCas9-SunTag-DNMT3A	DNA methylation (multiple copies)	[130]
	CRISPRoff	DNA methylation, histone methylation, and deacetylation (contains ZNF10 KRAB, DNMT3A, and DNMT3L protein domains)	[131]
Epigenetic activation	dCas9-p300	Histone acetylation	[120]
	dCas9-TET1CD	DNA demethylation	[125]

**Table 3:** Comparison of Cas9-derived proteins with regular SpCas9.

	CRISPR/Cas9	Base Editors	Prime Editors	Cas-CLOVER	Cas-FokI
DNA catalytic domain	RuvC and HNH (nuclease)	Cytidine deaminase or adenine deaminase	Cas9 H840A nickase, reverse transcriptase, and cellular endonuclease	Nuclease domain from *Clostridium* Clo051	FokI
DNA recognition	sgRNA (crRNA in complex with tracrRNA)	sgRNA (crRNA in complex with tracrRNA)	pegRNA (containing a primer binding site and a donor template)	Left and right sgRNA	Left and right sgRNA
Mechanism	Double-strand breaks in edited DNA (repaired by non-homologous end joining, NHEJ, or homology-directed repair, HDR)	Single-base changes (C to T and A to G) in edited DNA without introducing double-strand breaks	Accurately introduces small targeted insertions into the edited DNA sequence and removes and replaces bases	High-fidelity site-specific nuclease that introduces double-strand breaks in edited DNA with undetectable off-target activity	High-fidelity site-specific nuclease, that introduces double-strand break in edited DNA with undetectable off-target activity
Specificity	Tolerates mismatches	Tolerates mismatches	Tolerates mismatches	Enhanced specificity	Enhanced specificity
Ease of delivery	Easily delivered using multiple techniques	Harder to deliver due to the increased size	Harder to deliver due to the increased size	Harder to deliver due to the increased size	Harder to deliver due to the increased size
Limitations	Possible off-target effects (especially for SpCas9)	Narrow range of editing in the immediate vicinity of the PAM, potential off-target effects	Overall complexity of the system	Difficult to deliver (size)	Difficult to deliver (size)
Multiplexing	Easy	Easy	Easy	Easy	Easy
Active clinical trials	Yes (64/130)	Yes (4/130)	No	Yes (2/130)	No
Use in academic laboratories	26,717 results in PubMed^®^ (https://pubmed.ncbi.nlm.nih.gov/, accessed on 2 November 2023 with search term “CRISPR Cas9”	12,373 results in PubMed^®^ (https://pubmed.ncbi.nlm.nih.gov/, accessed on 2 November 2023 with search term “CRISPR Cas9 base editing”	306 results in PubMed^®^ (https://pubmed.ncbi.nlm.nih.gov/, accessed on 2 November 2023 with search term “CRISPR Cas9 prime editing”	3 results in PubMed^®^ (https://pubmed.ncbi.nlm.nih.gov/, accessed on 2 November 2023 with search term “Cas-CLOVER”	14 results in PubMed^®^ (https://pubmed.ncbi.nlm.nih.gov/, accessed on 2 November 2023 with search term “FokI-dCas9”
First time mentioned	2011	2016	2019	2020	2014

**Table 4:** CRISPR/Cas9 delivery strategies.

Delivery Approach	Mode of Cas9 and Guide RNA Delivery		
	DNA	mRNA	RNP
Electroporation	+	+	+
Viral vectors	+	+	−
Lipofection	+	+	+
Lipid nanoparticles	−	+	+
Polymer nanoparticles	−	−	+
Hydrogel nanoparticles	−	−	+
Gold nanoparticles	−	−	+
Graphene oxide	−	−	+
Metal−organic frameworks	−	−	+
Black phosphorus nanosheets	−	−	+
Cell-penetrating peptides	−	−	+
DNA nanostructures	−	−	+

**Table 5:** Comparison of different CRISPR/Cas and Fanzor protein properties.

	CRISPR/Cas9	CRISPR/Cas12	CRISPR/Cas13	CRISPR/Cas14	Fanzor Proteins
DNA catalytic domain	RuvC, HNH	RuvC-like nuclease domain, Nuc-domain	HEPN domains	RuvC	RuvC-like nuclease domain
Target	Double-stranded DNA	Double-stranded DNA	Single-stranded RNA	Single-stranded DNA	Double-stranded DNA
Collateral activity	No	Yes	Yes	Yes	No
DNA recognition	sgRNA (crRNA in complex with tracrRNA)	crRNA	crRNA	crRNA and tracrRNA	fRNA or ωRNA
PAM requirements	NGG, NAG for SpCas9, and other PAM variants for Cas9 orthologs (for details, see Table S4)	TTTN, TTTV (V = G, C, or A) For AsCpf1 from *Acidaminococcus* or LbCpf1 from *Lachnospiraceae*, and other PAM variants for Cas12 orthologs [362,363,364,365,366,367,368]	Requires protospacer flanking sequence–A, U, or C	None	Target adjacent motif preference is diverse, with a GC preference observed for the viral Fanzor proteins and AT preferences for the eukaryotic Fanzor proteins
Specificity	Regular SpCas9 tolerates mismatches, but high-fidelity variants exist	Cas12a has been successfully used for gene editing in vivo without any deleterious off-target effects	RNA-editor, no damage to DNA may occur	Cleaves ssDNA with high fidelity-sensitive to even a single mismatch in the target sequence	Needs further investigations
Ease of delivery	Easily delivered using multiple techniques	Easily delivered using multiple techniques	Easily delivered using multiple techniques	Easily delivered using multiple techniques	Needs further investigations
Limitations	GC-rich DNA targets Possible off-target effects (especially for SpCas9)	AT-rich DNA targets	Needs to be constitutively expressed to maintain the editing effect	Targets ssDNA	Unknown
Multiplexing	Easy	Easy	Easy	Easy	Possible
Active clinical trials	Yes (64/130)	Yes (2/130)	No	No	No
Use in academic laboratories	26,717 results in PubMed^®^ (https://pubmed.ncbi.nlm.nih.gov/, accessed on 2 November 2023 with search term “CRISPR Cas9”	1684 results in PubMed^®^ (https://pubmed.ncbi.nlm.nih.gov/, accessed on 2 November 2023 with search term “CRISPR Cas12a or Cpf1”	373 results in PubMed^®^ (https://pubmed.ncbi.nlm.nih.gov/, accessed on 2 November 2023 with search term “CRISPR Cas13”	28 results in PubMed^®^ (https://pubmed.ncbi.nlm.nih.gov/, accessed on 2 November 2023 with search term “CRISPR Cas14”	4 results in PubMed^®^ (https://pubmed.ncbi.nlm.nih.gov/, accessed on 2-November 2023 with search term “Fanzor”
First time mentioned	2011	2017	2017	2018	2013, 2023

## Data Availability

Not applicable.

## Supplementary Materials

The supporting information can be downloaded at: https://www.mdpi.com/article/10.3390/ijms242216077/s1.
